# Frontiers in Microfluidics, a Teaching Resource Review

**DOI:** 10.3390/bioengineering6040109

**Published:** 2019-12-03

**Authors:** Jack Merrin

**Affiliations:** Institute of Science and Technology Austria, Am Campus 1, 3400 Klosterneuburg, Austria; jack.merrin@ist.ac.at

**Keywords:** microfluidics, bioMEMS

## Abstract

This is a literature teaching resource review for biologically inspired microfluidics courses or exploring the diverse applications of microfluidics. The structure is around key papers and model organisms. While courses gradually change over time, a focus remains on understanding how microfluidics has developed as well as what it can and cannot do for researchers. As a primary starting point, we cover micro-fluid mechanics principles and microfabrication of devices. A variety of applications are discussed using model prokaryotic and eukaryotic organisms from the set of bacteria (*Escherichia coli*), trypanosomes (*Trypanosoma brucei*), yeast (*Saccharomyces cerevisiae*), slime molds (*Physarum polycephalum*), worms (*Caenorhabditis elegans*), flies (*Drosophila melangoster*), plants (*Arabidopsis thaliana*), and mouse immune cells (*Mus musculus*). Other engineering and biochemical methods discussed include biomimetics, organ on a chip, inkjet, droplet microfluidics, biotic games, and diagnostics. While we have not yet reached the end-all lab on a chip, microfluidics can still be used effectively for specific applications.

## 1. Introduction

This review closely follows how microfluidics is taught in my course. The course usually consists of twelve lectures and six practical sessions aimed primarily for biology graduate students. My favorite textbook for biologically inspired microfluidics is Folch’s Introduction to bioMEMS. There is broad coverage of topics and an extensive collection of color figures from the literature [1]. There exist other good books on biophysics which can be used in conjunction like the Physical Biology of the Cell, which is also recommended for similar reasons as Folch’s book [2]. My way of organizing the course is to consider the physical principles and microfabrication techniques in the first two classes. The next classes address diverse topics in microfluidics applied to biology. It may be beneficial to arrange a course where students present their own project ideas at the end. Designing a project tests whether students have mastered the material and if they are ready for research. It is usually not a massive investment to try new microfluidics research projects once a lab is already setup. Hopefully, this review can help others prepare their courses or discover new avenues of research in microfluidics.

## 2. Physical Principles

Microfluidic physics and engineering principles are necessary to go from concept to device and predict their operation. Fluid mechanics is described most generally by the Navier–Stokes equation.

$$\rho \frac{\partial v}{\partial t}+\rho \left(v\cdot \nabla \right)v=-\nabla p+\eta \nabla^{2}v+\rho g$$

An analytic turbulent solution to the Navier–Stokes equation can win you a million dollar millennium prize. Solving nonlinear partial differential equations is notoriously difficult.

Physical length scales encountered in microfluidics allow the Navier-Stokes equation to be often approximated. This occurs when the Reynolds number Re is small.

$$Re=\frac{\rho LV}{\eta }\;0=-\nabla p+\eta \nabla^{2}v+\rho g\;Re<<1.$$

This linear partial differential equation can actually be solved for simple situations such as flow through a horizontal cylindrical pipe of radius *R*. The velocity profile is parabolic and largest in the center of the pipe.

$$v\left(r\right)=v_{max}\left(1-r^{2}/R^{2}\right)\;\Delta p=\frac{8\eta LQ}{\pi R^{4}}.$$

Hagen–Poiseuille flow is reminiscent of Ohm’s law, V=IR. The flow rate *Q* corresponds to electric current *I*. A pressure difference Δp corresponds to voltage. The remaining terms are the hydraulic resistance.

The main difference in microfluidics is that the hydraulic resistance scales as one over the cross-sectional area squared compared to a similar calculation with the resistivity of a wire.

$$R_{h}\propto \frac{\eta L}{{\left(\pi R^{2}\right)}^{2}}\;R_{OHM}=\frac{\rho L}{A}.$$

Usually, one manufactures rectangular pipes where the hydraulic resistance follows a more complicated expression that depends on the aspect ratio. Fluid dynamics simulators such as COMSOL are often the final word, but you can get pretty far conceptually with electronic circuit analogies.

Flows in microfluidics are laminar or have static streamlines. Flow velocities are higher near the center of a channel and zero at the walls. Taylor dispersion is when velocity gradients spread out a flowing fluid, which has consequences for mixing by diffusion. Fluid can be transported as a plug via water in oil droplets or using electric fields.

Conservation of mass flowing through a pipe gives the continuity relationship

vA = const.

If the cross-section of a continuous pipe shrinks, the average velocity goes up.

Archimede’s principle is another basic fluid concept, which states that objects have a buoyant force equal to the quantity of liquid displaced. However, cells often have a similar density to water.

One consequence of life at low Reynolds number is the scallop theorem [3]. Scallops only move because of turbulence. On the ultra-high viscous scale, it is not possible to swim forward because forwards and backward strokes provide no net movement. Bacteria move at low Reynolds number by a rotating corkscrew flagellum that breaks the symmetry.

Chemical diffusion and thermal transport often dictate microfluidic designs. The chemical diffusion equation and heat transfer equation are similar.

$$\frac{\partial c}{\partial t}=D\nabla^{2}c\;\frac{\partial T}{\partial t}=k\nabla^{2}T.$$

Also of interest is Fick’s equation describing the flux from a concentration gradient.

J=−D∇c.

The primary way of mixing two different fluids is through diffusion since there is no turbulence at low Reynolds number. Mixing due to flows versus diffusion is described by the Peclet number.

$$Pe=\frac{Lv}{D}.$$

The spin-coated thickness of silicone is by the Emslie, Bonner and Peck’s equation equal to

$$h\approx \sqrt{\frac{3\eta }{4\rho }}\times \frac{1}{\omega \sqrt{t}}.$$

This result is independent of the initial height profile provided the rotation speed or spin time is long enough. Photoresist spin-coating usually follows some power-law version of this equation due to solvent evaporation during spinning or baking.

Other practical concepts include hydrophobicity, hydrophilicity, and contact angle to evaluate how a liquid wets a material. More mathematically inclined students should be directed to “Microfluidics: Fluid Physics at the Nanoliter Scale” or books on fluid mechanics [4].

## 3. Microfabrication

**Figure 1:**
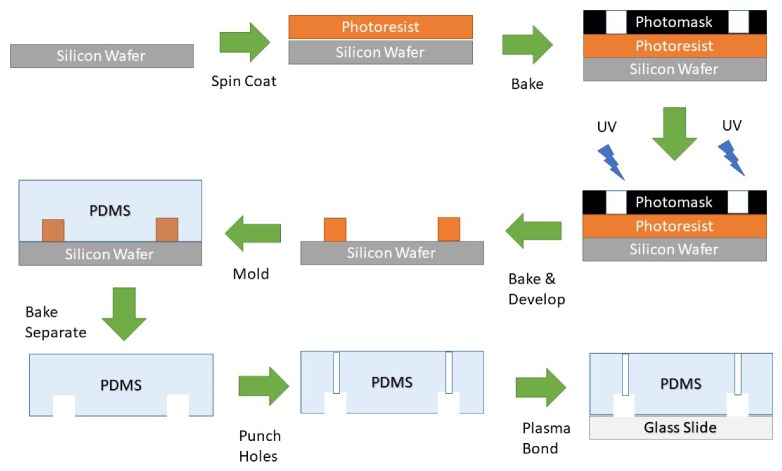
The basic procedure to create a microfluidic device with negative SU8 photoresist. Spin coating, baking, UV exposure through a photomask, molding with silicone, punching microchannel ports, and bonding polydimethylsiloxane (PDMS) to glass with plasma.

Microfluidics borrows methods from microelectronics to engineer devices. Structures wider than the wavelength of light can be made with photolithography. Microstructures can be replicated into a microfluidic device through a procedure called soft lithography, which is described in Figure 1 [5,6].

In photolithography, one starts with a flat substrate, such as glass or a silicon wafer. The substrate is spin-coated with photoresist. Then UV light is shined through a photomask onto the photoresist. Positive photoresists can be developed away where there was UV exposure, while negative photoresists harden where exposed. The development leaves the substrate with a surface profile and pattern.

Liquid silicone, polydimethylsiloxane (PDMS) is baked on top of the master mold and then peeled off. PDMS is an ideal material for academic research because it is inexpensive, transparent, elastic, and biocompatible. The silicone bonds to glass slides with an air plasma treatment. One can repeat this procedure for arbitrary patterns according to your application.

The entire microfabrication procedure from concept, drawing, photomask, microfabrication, and PDMS device can be done in a day (rapid prototyping) if one can obtain the photomask locally. Otherwise, it usually takes several days to obtain the photomask from a supplier. A CAD (computer-aided design) program is used to draw the photomask at 1:1 resolution. Standard software for this is AutoCad, but other software like Coreldraw or Adobe Illustrator are easier to use with file converting software such as LinkCad. Photomask companies usually require the design to be converted into an exact file type, such as DXF, GERBER, or GDS2. EPS and PDF files are not precise at one-micron resolution. Chrome on glass photomasks can go down to about 1-micron resolution while transparency photomasks are only accurate to 10-micron resolution.

The most robust photoresist is a negative photoresist called SU8, a chemical based on eight epoxy structures. SU8 is the best for vertical side wall structures that are chemically and mechanically resistant. There are modern SU8 6000 TF series photoresists that are easier to work with in terms of adhesion and are less prone to thermal cracking. One can usually remove cracks in the older SU8 chemicals by heating at 135 °C for five minutes.

Silicon wafers or glass slides can be passivated with (tridecafluoro-1,1,2,2-tetrahydrooctyl)-1- trichlorosilane for easy PDMS surface release. The procedure is to evaporate 10 microliters in a desiccator under a sealed vacuum for one hour to coat a wafer. Changing the PDMS curing ratio from 10:1 to 3:1 would result in permanent bonding without silanization. Silanes are very corrosive and should be used in a fume hood and away from water.

The best way to connect to a PDMS microchannel is with steel tubing, which can be made by cutting and sanding syringe needles. The steel tubing seals on one end to holes punched in the PDMS (0.5 mm biopsy punches Darwin microfluidics) and the other end seals to Tygon tubing, for example. Luer lock dispensing needles interface directly with peristaltic pumps, syringe pumps, pressurized bottles (always use pressure safe bottles below 1 atm), or a gravity flow-based pressure source.

**Figure 2:**
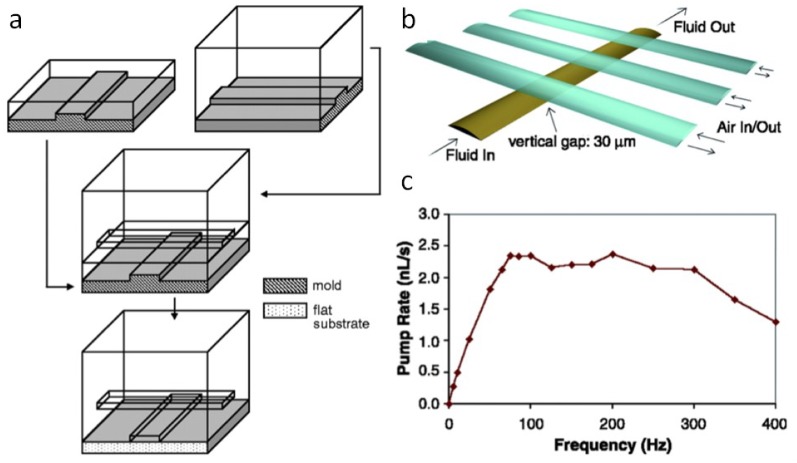
(**a**) The multilayer soft lithography procedure is performed with two master wafers to produce channels in several layers with a thin membrane between them. (**b**) Microfluidic valves made with rounded channels in a sequence of three can be alternately pressurized and used as a peristaltic pump. (**c**) The average flow rate of the peristaltic pump in panel B as a function of valve actuation frequency. The pump rate saturates at high frequency because of the few millisecond time scale of the valves to recoil to their original states [7].

Microfluidic valves can be made with two crossing channels in different layers with a PDMS membrane between them. Two layers with different curing ratios 20:1 and 5:1 can be bonded together in an early stage of curing. When the control channel is filled with water (avoids air bubble injection) and pressurized, it presses on the rounded fluid channel and seals it. This valve can be used to redirect flows or as part of a peristaltic pump as in Figure 2. The fluid channel has to be rounded to seal completely, like squashing a coke can with no ends. If the fluid channel is rectangular at the valve, leaks on the sides create a sieve valve.

**Figure 3:**
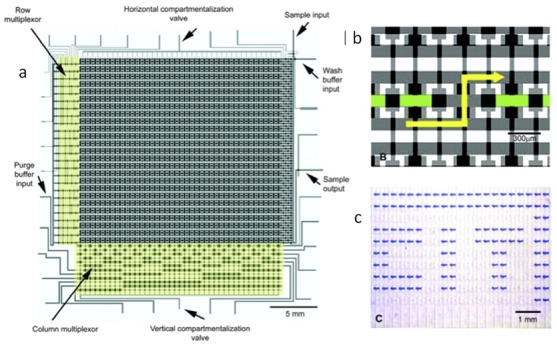
(**a**) Microfluidic RAM or fluid display. (**b**) Valve topology necessary for storing and exchanging fluid in the display pixels. (**c**) The actual devices showing pixels storing blue dye versus water everywhere else forming the initials of the California Institute of Technology [8].

Rounded masters can be made with positive photoresists that reflow at high temperatures. When developing the procedure for multilayer microfluidic valves, RTV 615 brand PDMS should be used instead of Sylgard 184 and shrinkage of 1.5 percent when going from 80 °C to room temperature should be taken into account. Operating microfluidic valves can get quite technical, but the rewards are often highly functional integrated devices as in Figure 3 [7,8].

For some other technical nuances of working with microfluidics, the Chips and Tips blog is a good resource. Madou’s three-volume set also covers pretty much anything you can imagine to fabricate in the cleanroom if a more technical procedure is needed [9].

## 4. Overview

This review will primarily focus on biological and medical applications of microfluidics. Microfluidics is also relevant to physics, chemistry, and engineering. Microfluidics is an experimental method to advance some scientific aims. In this sense, we try to predict and control how physical entities behave and interact. In terms of biology, microfluidics applies to cell culture, physiology, medicine, model organisms, drug screening, behavioral analysis, biochemistry, and toxicology. From a physics and chemistry point of view, microfluidics is used to control thermodynamics quantities and chemical reactions in small liquid volume experiments. From a biotechnology perspective, microfluidics is used to miniaturize assays and equipment for diagnostics, biomimetics, scientific instrumentation, commercialization, or manufacturing processes. The types of microfluidic devices typically sort into classes, which include single-layer devices, valve-based devices, droplet-based devices, and other novel methods like paper-based microfluidics. There are numerous microfluidic topics such that they cannot all comprise a single review. I will try to emphasize and state why the themes I have chosen are relevant, microfluidics is needed, and the scientific principles they elucidate.

## 5. Bacterial Microfluidics

Even for growing bacteria, microfluidics can already be quite useful. In a traditional cell culture experiment, bacteria go through phases of growth depending on their history, the depletion of nutrients, and the accumulation of waste products. Growing cells on a petri dish or in a flask do not result in the same conditions over time. One can imagine growing cells in a microfluidic system with an exchange of nutrients and depletion of waste products for as long as possible by diffusion, using membranes, or circulation of media and cells. Cell culture is a good entry point into the applications of microfluidics. How can we grow cells better?

**Figure 4:**
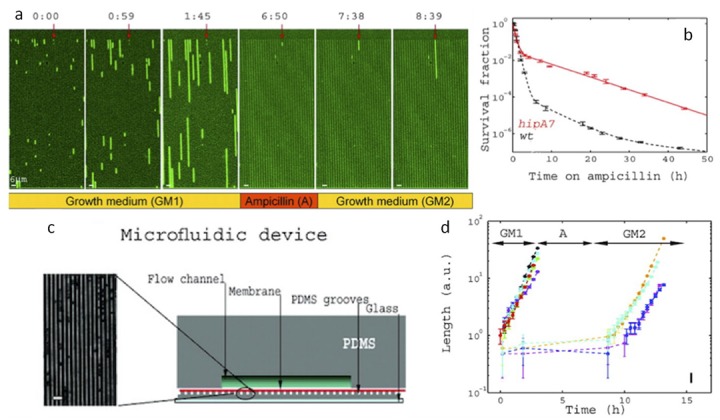
(**a**) Bacterial lineages are growing in alternating applications of ampicillin in linear channels with a width of one micron. (**b**) The number of surviving bacteria colonies after limited exposure to ampicillin does not decay completely exponentially due to the existence of rare persister cells. (**c**) Microfluidic flow cell with an integrated membrane for rapidly changing the environment and sealing cells in the linear channel. (**d**) Normal cells grow exponentially, while cells in the persister state are slow-growing. Persister cells randomly switch back to the normal state with a normal growth rate even after an intermediate exposure to ampicillin [10].

In the first paper, we consider *Escherichia coli* cells grown in one-micron wide channels sealed by a semipermeable membrane to a flow cell as in Figure 4 [10]. Flowing different media allows for rapid environmental changes, and cell lineages accumulate in the channels in one layer. In a classical petri dish experiment, cells forming a colony pile up on top of each other, and it is hard to track the layers on the microscope. Using time-lapse microscopy and alternating environmental phases of media or media containing ampicillin reveals persister cells in a bacteria population. The persister cells are a non-growing or slowly growing phenotype, but they can transition into the normal growth phase at a later time. Ampicillin affects only actively growing cells while they make membrane-stabilizing structures. The persister cells avoid the selection by an antibiotic, but normal cells are susceptible because they grow at a normal rate in the presence of ampicillin. Due to persistence, certain infections are incurable with normal antibiotics. Other medicines must be created that target the persistent state.

**Figure 5:**
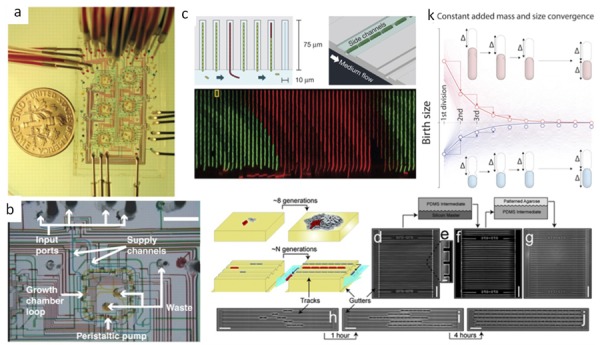
(**a**) Six microchemostats in parallel filled with dye illustrating fluid and valve layers [11]. (**b**) Internal workings of the self-cleaning microchambers [11]. (**c**) A mother machine can be used to investigate phenotypic switching and nongenetic individuality [14]. (**d**–**j**) Agar based mother machine with both ends of the device open to flow [17] (**k**) A new model of cell division by adding a constant volume with each division based on mother machine experiments [13].

Another traditional way to grow bacteria is in a chemostat, which is a device that mixes in the fresh medium into a culture vessel and dilutes out an equal amount. The bacteria then reach a steady-state density in the device until a new mutant comes along that grows faster and takes over. Chemostats typically operate at low cell density. Common problems with chemostats are biofilms and cells sticking to the walls. The same problem exists when you shift down to the microfluidics scale. Balagadd et al. [11] developed a self-cleaning microchemostat where partitions of the volume are diluted, sterilized, new media is introduced, and the new media is mixed into the system as in Figure 5a,b. It was also observed that unstable systems biology constructs could be studied for longer in microfluidics before evolution eliminated them. This was the most advanced microfluidic device at the time and a beautiful illustration of what can be done with valve microfluidics.

One of the most exciting developments in microfluidics for bacteria is called a mother machine [12]. A mother machine is a one-micron wide channel with one end closed and a taller feeding channel on the other side as in Figure 5c. The mother cell holds its position, and cell lineages are emitted out of the channel. In the original paper, they settle the question of whether bacteria are immortal or not. This microfluidic configuration also takes away excess cells so that you can still study individual cells on the microscope for many days without them piling up. After roughly a hundred generations, the mother cell accumulates an old pole with cellular damage. The cell then either dies or filaments such that it does not continue dividing. The new daughter cells keep growing normally. This process can be studied in different strains and with or without an SOS repair system to see how bacteria age. By changing the flowing media, you can rapidly shift environments and see the effects on single cells. Shifting up and down rapidly in chemical environments is not possible in a chemostat without diluting everything, but it is possible in a mother machine.

Another exciting application of the mother machine is to study how cells maintain cell size as in Figure 5k. By careful studies in different nutrients, it was discovered that bacteria like *E. coli* always add a constant volume then divide in the middle of the cell [13]. This allows the total length to regress to the mean if the cells start too big or too small.

The use of the mother machine reveals the phenotypes of single-cells and their progeny. Bacterial phenotypes can vary from cell to cell even if they contain identical DNA. What controls this heterogeneity is of biological interest. In *Bacillus subtilis*, the decision to be solitary cells, motile cells, or chained sessile cells has been investigated [14]. For *E. coli*, the unequal partitioning of drug efflux pumps during cell division can lead to phenotypic heterogeneity among cells [15]. In another study, mother machine study, genetic circuits responsive to light allow computer feedback to shape the phenotypic population characteristics [16]. The use of the mother machine has become a fruitful research area, and we expect many discoveries to be made with this system.

Agar is a nutrient baring hydrogel that allows diffusion. Agar microfluidic devices are possible instead of silicone. An agar version of the mother machine open on both ends was made as in Figure 5d–j [17]. One can control the chemical environment of cells by diffusion in such a system. Cells in adjacent channels can also chemically communicate through diffusion. Most bacteria in soil cannot be cultured individually because the community provides necessary nutrients. Perhaps this system can further elucidate the viable but non-culturable problem in microbiology.

*E. coli* usually swim parallel to walls until they tumble. A system of funnels can then concentrate bacteria in separate chambers in the absence of chemotactic gradients [18]. Such devices may be possible to screen for motility mutants, separate bacteria, or accumulate high densities of bacteria in a microfluidic device.

A classic experiment with bacteria is to alternate cycles of glucose and lactose. Bacteria typically only utilize glucose before lactose. Oscillations of sugar environments reveal a new type of memory adaptation where cells use both sugars [19]. Microfluidics allows one to distinguish between analog and digital phenotypes at the single-cell level.

Microfluidics has also been used to do high throughput analyses of bacterial strains on the microscope. With fluorescent reporter strains of some thousand different genes, the number distribution of proteins in the proteome of *E. coli* was measured [20]. Measurements were facilitated by arranging many channels on a chip where different strains could be injected one after another without tedious changing of individual microscope slides. The paper concludes that proteins are generally gamma-distributed in a population due to bursts of protein expression.

## 6. *Saccharomyces cerevisiae* Microfluidics

Saccharomyces cerevisiae or baker’s yeast is another model organism. Culturing eukaryotic yeast with microfluidics is useful for similar reasons for bacteria. Different implementations of chemostats exist in microfluidics [21]. One way to perform the dilution is to have a main chamber with tiny side outlets that allow diffusion, but through which the cells cannot pass. In an extra vertical layer, channels flowing water can heat or cool the device. Mother machines and microfluidic valves have since taken over as a better way to grow in continuous exponential growth conditions.

**Figure 6:**
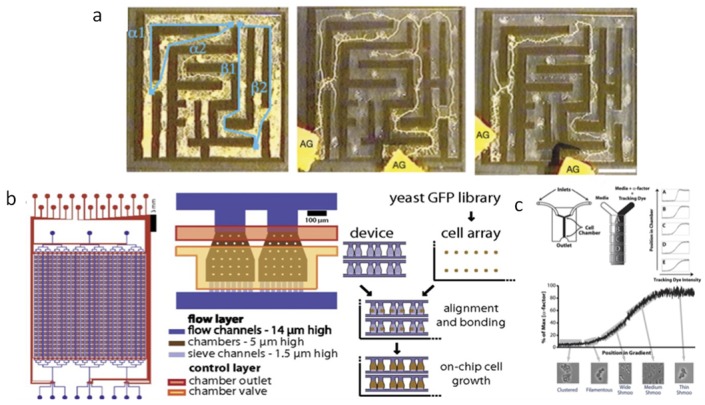
(**a**) Physarum initially fills a microfluidic maze (first panel) then beings to solves the microfluidic maze between two food sources (second panel). The final solution is optimized for the shortest path (third panel) [29]. (**b**) Massively-in-parallel microchemostats for proteomics investigations into multiple strains [23]. (**c**) Gradient generating device for studying yeast mating patterns [22].

It is possible to generate chemical gradients in microfluidic devices under flow. There are two mating types of yeast, and they emit hormones to signal to each other. Yeast can make a protrusion called a schmoo, which allows them to conjugate according to the local mating factor concentration. By flowing a gradient of the mating hormone under attached cells, the molecular details and quantitative behavior of schmoos has been measured as in Figure 6 [22]. Older pipette-based assays are not as quantitative and reproducible as measurements taken with microfluidic gradient devices. This is an example of finding a biological problem that matches a microfluidic capability.

In a recent paper, 1152 different strains of yeast were grown in a single microfluidic device under chemostat conditions as in Figure 6 [23]. This allows proteome analysis in live cells. With microfluidics, one can also change the environments of the cells, which will enable dynamic and time series proteome analyses versus gene chips, which are only a snapshot and average populations.

## 7. *Trypanosoma brucei*

Trypanosomes are parasitic protozoa with dimensions of roughly three microns by twenty microns. They affect many mammalian species and are best known for causing African sleeping sickness. Hochstetter et al. investigated the effects of 2-deoxy-D-glucose and suramin on trypanosomes in microfluidic devices [24]. They study how these drugs kill the parasites or halt their growth. They also study how cell motility and morphology vary as a function of drug concentration. Often other methods such as optical tweezers work well with microfluidics. One can then have chambers and channels where a reproducible environment can be changed or maintained stably for long periods of observation. In a series of recent biophysical measurements, the propulsive force, dissipative energy, and power generation of trypanosomes were measured [25]. The results depended on whether trypanosomes had one or two flagella before they were about to divide. The optical trap does not excessively heat the cells and can be used to visualize running and tumbling motions in conjunction with a microscope.

Another addressable with microfluidics is the detection of blood parasites such as trypanosomes. The usual threshold for microscope identification of such a pathogen is 10,000 per milliliter. But trypanosomes can cause disease at as low as 100 per milliliter. Deterministic later displacement (DLD) is a microfluidic sorting method that can enrich trypanosome concentrations. In this method, a series of rows with successive shifts can be tuned to sort particles of various sizes. DLD has been demonstrated to sort white blood cells from red blood cells for example, or sort trypanosomes from red blood cells [26]. This device is simple to operate via a disposable syringe. Such cheap and easy to operate chips may find use in the developing world. There are several reviews on how deterministic lateral displacement work for particle and cell separations [27,28]. It is trivial to mix, but nontrivial to unmix.

## 8. *Physarum polycephalum*

*Physarum polycephalum* is a slime mold that can move on agar surfaces and likes to feed on oats. Physarum is of interest because it has an impressive intelligence to solve spatial optimization problems, yet lacks a brain. Physarum changes shape depending on nutrient locations by extending pseudopodia. Physarum’s body solves the shortest distance path through a microfluidic maze with oats at the entrance and exit as in Figure 6 [29]. Physarum also solves the optimal way of connecting food sources representing a Japanese train railway map [30].

## 9. *Caenorhabditis elegans* Microfluidics

*Caenorhabditis elegans* or *C. elegans* is a well-studied model eukaryotic organism for developmental biology and neuroscience of intense interest. Each worm grows the same number of 959 somatic cells and 302 neurons in a specific arrangement and lineage.

There are several ways that *C. elegans* research benefits from microfluidics procedures. One can image worms because PDMS is transparent. Worms are motile, so it is difficult to track them and image them. Microfluidic immobilization works by pushing worms into a channel that exactly fits their size, clamping them with a microfluidic valve, chilling them with circulating 4° Celsius water, vacuum suction on the worms, or applying anesthetics. Surgery to ablate neurons after worms are immobilized is possible. One can make chambers where worms’ entire lifespan can be tracked in a microchamber. Worms can be sorted based on several factors such as morphology, neural activity, or gene activity at the rate of about 100 per hour to also find mutants. Worms can also be exposed to chemicals for drug screening and toxicology. Worms can be studied in behavioral assays on the chip, for example, how they move in pillar arrays or classic chemotaxis experiments.

**Figure 7:**
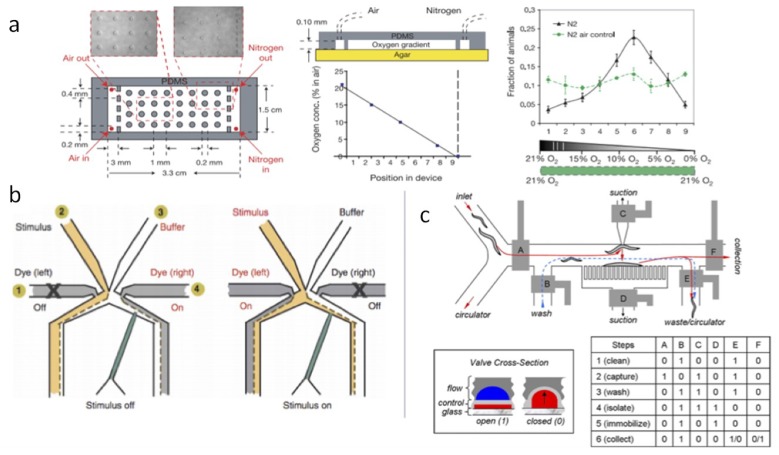
(**a**) Microfluidic oxygen concentration generating device. Worms accumulate at an optimal oxygen concentration [31]. (**b**) Microfluidic device for olfaction and neuroscience experiments on immobilized worms [32]. (**c**) High throughput worm sorter, imaging, and surgery chip [33].

In the first microfluidic application to the study of worms, an oxygen gradient was set up inside a microfluidic device as in Figure 7a [31]. It was found that worms are attracted to an optimal concentration between zero and the normal 21 percent. It is not clear why this is the case. Perhaps, this may be a social feeding behavior or keep worms in dirt at a certain depth away from the surface. In a later study, worms were immobilized with their heads extending into a microfluidic channel. Then different stimulants can flow over the nose of the animal as in Figure 7b. Since the worm was immobilized, fluorescent tracking of neurons can also be coordinated with the stimulus [32].

One early paper made some progress is high throughput surgery, sorting, and cultivation of worms in microfluidics using valve microfluidics as in Figure 7c [33]. This allows the isolation of interesting mutants for further analysis and automation of tasks that would be too tedious if done manually in the lab. Worms may spend their entire lives in microfluidic devices where a large amount of data can be accumulated [34]. There is a recent review of worm research with microfluidics by Kamili et al. [35].

## 10. *Drosophila melangoster* Microfluidics

*Drosophila* is also an important model system in developmental biology. To perturb the natural segmentation development patterns of embryos a novel type of microfluidic device was developed [36]. Half the embryo was exposed to a cold shift, and the other half was hot. It would be difficult to set up this kind of thermal gradient without microfluidics. Any perturbation to the normal segmentation development patterns is of interest to see how robust flies are. Other practical studies with flies involve trapping embryos in a chamber with a valve or making embryo sorters with microfluidic valves [37,38]. Normally flies are not studied so much with microfluidics, but the future will reveal if these methods are useful.

## 11. *Arabidopsis thaliana* Microfluidics

*Arabidopsis thaliana* is the primary plant model organism for its small size, fast life cycle, developmental biology, genome sequencing, and the ability to genetically manipulate it. Recently there have been several papers where root growth as a function of modulating environments can be studied using so-called microfluidic Rootchips [39,40]. Microfluidics is useful for gaining physical and chemical control of the root environment while still imaging the roots. It is advantageous to track roots in microfluidics rather than having one time snapshots by pulling them out of the dirt. Traditional hydroponics is also incompatible with microscopy. Microfluidic valves can be used to modulate the environment of the roots, which is not simple with standard culture methods.

**Figure 8:**
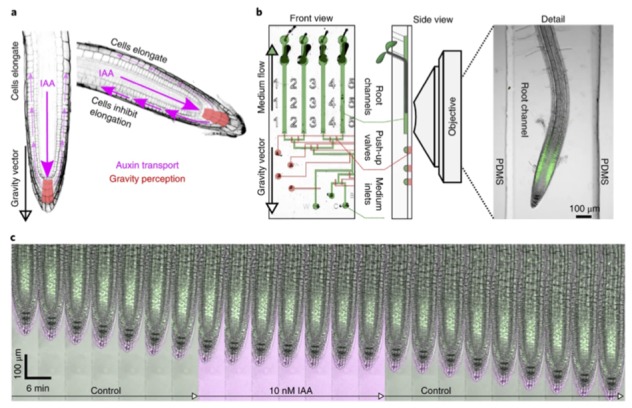
(**a**) Auxin hormone regulates root tip growth and is involved in gravitropism. (**b**) RootChip microfluidic device for alternating environments. Growth or root into the microfluidic channel. (**c**) Root tip growth halts almost instantaneously when auxin is applied [40].

In our study, we find that auxin can rapidly modulate root tip growth, even in a few seconds, which is faster than transcriptional timescales as in Figure 8. Auxin is an important hormone also involved in gravity sensing. Many of the molecular details of this process remain to be discovered. The theory of plant gravitropism hypothesizes that starch granules sink to the bottom of root tip cells where other unknown proteins there modulate local auxin concentration. Auxin, in turn, modulates the direction of root growth.

A useful review of plant microfluidics is also available which discusses applications to pollen and seeds [41]. A new frontier in this field is the so-called dual flow Rootchips. Laminar flows on several sides of the root allow one to see which processes respond locally versus globally [42].

## 12. Immunology and Chemotaxis

There are many types of investigations of chemotaxis with microfluidics. Standard methods of gradient generation include combining different laminar streams in a flow and then letting diffusion average the steps in the stream to produce a linear gradient. A novel agarose based microfluidic device can be used for chemotaxis studies including immune cells [43]. It contains an agar barrier that allows the creation of gradients by diffusion without a required flow that may wash cells away. A pre-established gradient can be generated in the agar so that when one channel is recirculated, the gradient can be established quickly by diffusion through the agar back inside it.

An alternative method to establish gradients without flow is to use a microfluidic channel between two microvalves. The valves can open quickly, allowing some diffusion and then can close to keep the gradient. The concentrations can be replenished behind the valves, and the process can be repeated. In a recent study, this method allowed the investigation of competition between surface-bound chemoattractants and diffusible chemoattractants in immune cells [44].

In a study by Love et al., they produce a PDMS array of microchambers on a microscope slide [45]. Then immune cells that produce monoclonal antibodies are distributed in wells. Then the cells can be imaged and selected from the wells that produce the right antibody. The advantages of this system are that it is both high throughput and cheaper than traditional methods. Monoclonal antibodies are useful for biotechnology and medicine. Another exciting procedure described in this paper is how to see what sequence of molecules the cells emit over time by repeatedly replacing the glass slides covering the chambers coated with antibodies and analyzing them.

Enzyme-linked immunosorbent assay (ELISA) is an immunological biotechnique for detecting small concentrations of ligands using antibodies and fluorescent chemical reactions. With microfluidics, the sensitivity is increased by placing a valve over the detection region, thus reducing the background signal. The dynamic range of ligand detection is five orders of magnitude down to the tens of attomolar [46].

In organisms, immune cells routinely have to move through gaps that are smaller than the cells. Dendritic cells can extend protrusions to test choices between different trajectories with different gaps or pore sizes and choose the path of least resistance. This is advantageous to avoid damage to the nucleus when squeezing through narrow pores. In a recent study, dendritic cells are made to move through various microfluidic four-way junctions and mazes with different pore sizes to study this behavior [47].

## 13. Biomimetics

Biomimicry is taking inspiration from living organisms to design engineered products. Much of the inspiration comes from the micro-level or cellular level. Microfluidics can be one way to approach biomimicry. Geckos have the unusual ability to stick to the walls and ceiling; micro pillar-like structures on the feet called setae create a strong Van der Waals interaction with surfaces. Scientists try to mimic these structures in the lab to make adhesives and tape. The laboratory versions so far are not repeatable or do not work in wet environments. In a study, Lee et al. found that if they used a molecule from sea mussels, they could improve the gecko style adhesion even underwater. Practical applications include surgery bandages or manufacturing wet tolerant adhesives [48].

Similarly, organisms like an octopus also need to use adhesion underwater. They have evolved suction cups on their tentacles to grab objects. In a recent study, biomimetic suction cups were fabricated from PDMS and were also found to be able to adhere in dry or wet environments [49]. It is purely a physical interaction of the suction cup with the surface. Such suction cups can be useful in engineering.

## 14. Tissues and Organs on a Chip

There is an interest in generating microfluidic organs or tissues on a chip. The primary rationale is that humans cannot be experimented on directly. Animal models may not mimic human physiology. Having organs on a chip is also useful from a biotechnology point of view because it can reduce investments and time in drug discovery and drug testing. Whereas animal models take up a lot of space, organs on a chip can be miniaturized and run in parallel on a single chip. Traditional cell culture methods may not be indicative of in vivo human physiology. Cancer on a chip can also be used to test drugs in a disease model. Neural organoids and systems that mimic the blood–brain barrier are also being created. The realistic human model would be to have multiple organs on a chip working in conjunction as a body on a chip. Different organs send each other chemical signals, hormones, and metabolites. Several recent reviews give additional rationals for pursuing organs on a chip [50,51,52].

In one successful study, an artificial lung section on a chip system was constructed. It used a PDMS scaffold that could expand and contract to behave more like a breathing lung. It allowed endothelial and epithelial layers of cells to be cultured in the device. This system allows drug testing and toxicology analyses without sacrificing animals. The result of introducing contraction and expansion of the device using air pressure is that the system performed more like natural mice lungs in terms of immune response and nanoparticle uptake [53].

## 15. Inkjet

One of the most common applications of microfluidics that has been around for a long time is your inkjet printer. Inkjet is of interest because people want to print color photos for as cheap as possible. Envision the possibilities of printing biomolecules or cells instead of regular ink. In principle, one could dispense self-organizing tissues or organs. One limitation is the requirement of vascularization required for organs like a heart.

There are two styles of inkjet printheads. One contains a piezoelectric component that forces droplets out a nozzle. The second type is thermal, where air bubbles are heated and expand inside the nozzle ejecting droplets. Inkjet printing companies often sell the printers at cost, hoping to make profits selling the inks! Some recent inkjet cartridges have special chips that only allow a certain number of prints, regardless of whether ink remains. Protections can be problematic if you want to reverse engineer consumer electronics for your experiment. There are also bootleg ink reservoirs that are much more inexpensive or bypass the protection circuits. Femtoliter inkjet printers of the future may be involved in many manufacturing processes.

We have constructed a bacterial inkjet printer which can print regular arrays of cell colonies [54]. The interest of printing bacteria this way is to study their spatial interactions and chemical communication precisely. The number of cells per droplet is Poisson distributed. It is possible to dispense single cells, but typical drops spread out a bit when they hit the surface. It would be challenging to print cells at this fine detail and arrangement without a robot or inkjet printer. While thermal inkjet printheads are disposable, they heat shock printed cells. Piezoelectric printheads are not disposable and operate at room temperature. One should keep in mind that printheads can accumulate biofilms.

Inkjet printers have been used to replicate phosphoramidite chemistry that is used to generate DNA chips for profiling gene expression. The original POSAM (piezoelectric oligonucleotide synthesizer and microarrayer) paper demonstrated the capability to print custom gene chips for about eighty US dollars per slide [55]. Configurable gene chips are of interest because the original Affymetrix technology uses photolithography. Four photomasks for every nucleotide in the sequence get very expensive quickly for customization. Nowadays, Agilent has improved the POSAM system, and million spot custom gene chips can be purchased for around 500–1000 US dollars. Printing a gene chip and then extracting the DNA is one route to reducing the cost of DNA synthesis for synthetic biology [56,57].

## 16. Biochemistry

Biochemistry is the chemistry of living organisms and divided into metabolism, protein science, and molecular genetics. Microfluidics has the potential to study these subjects in vivo and in vitro. It is often possible to remake any of the hundreds of possible biochemical assays using microfluidics. Often microfluidics can be superior in biochemical assays in terms of costs, sensitivity, less required reagents, automation, and the speed of assays. Some typical biochemical assays that are amenable to microfluidics include quantification of concentrations, separation, purification, enzyme kinetics, ligand dynamics, toxicology, and drug screening.

**Figure 9:**
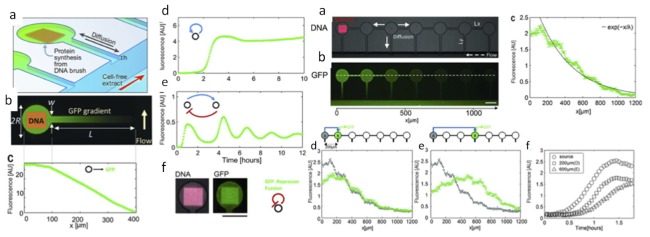
(**a-left**) Cell-free-extract artificial-development system in a microchannel with DNA template attached to the surface of the chamber. GFP expression in a constitutive system after four hours, image (**b-left**) and profile (**c-left**). Expression profiles for a positive feedback circuit (**d-left**) and oscillations in a circuit containing repression (**e-left**). (**f-left**) Image with GFP fused to repressor that binds to DNA. (**a**,**b-right**) Artificial development system with an array of chambers. (**c-right**) expression profile along n connected chambers. (**d**,**e-right**) profiles with GFP patterning in the second or fourth compartment. (**f-right**) kinetics of GFP expression [58].

A cell-free system is a lysate of bacteria containing all the functional enzymes, DNA templates, and ribosomes to perform gene expression. One might be interested in a cell-free system for toxic protein production, eliminating evolution from the equation in your experiment, or for artificial life experiments. Cell-free systems are ideal for testing synthetic biology circuits. In one recent paper, a DNA template attaches to the glass substrate of a chamber, and the cell-free lysate is allowed to diffuse as in Figure 9. This produces a kind of artificial geometrical development system where compartments communicate with one another biochemically [58]. Readouts of spatial gene expression patterns are done with green fluorescent proteins.

X-ray crystallography, an active field in biochemistry, is often used to determine the 3D structure of proteins or biomolecules. One limitation of crystallography is finding the right concentrations and pH of reagents to form crystals. Various microfluidic devices can determine micro-crystallization conditions in a combinatorial and parallel way. In a recent paper, protein structures were obtained from an array of microcrystals generated on a microfluidic chip [59]. The problem of X-ray damage is avoidable by testing thousands of individual crystals with short X-ray exposures.

Quantitative PCR (polymerase chain reaction) also benefits by scaling down to microfluidics. PCR can be used to quantify DNA concentrations, RNA concentrations, nucleotide polymorphisms, and forensics. PCR can be more accurate and sensitive by diluting the sample into millions of microfluidic chambers where concentrations approach one molecule per chamber. PCR can occur with a single molecule template with a dynamic range of $10^{7}$ in the experiment [60].

SELEX (systematic evolution of ligands by exponential enrichment) is a type of directed evolution experiment to design better functioning enzymes. It works faster than natural evolution, but many rounds of lab manipulations can be tedious. Microfluidics works as a good lab on a chip to automate, speed up the process, and reduce the use of reagents [61].

Another important aspect of biochemistry is the screening of drugs for desired effects on cells. The space of all chemicals is virtually infinite, so effective means of creating and high throughput screening of different compounds is crucial. Drugs are typically useful when they inhibit, interfere with, or repair a biochemical process.

One of the best ways to quantify biochemistry with microfluidics is to couple devices to mass spectrometry [62]. Another way to test many different types of drugs simultaneously is to optically barcode droplets that contain different drugs and then fuse them with droplets containing cells. Working with six fluorescent dyes allows up to $2^{6}=64$ possible barcodes. When the data is collected in parallel, one can backtrack with the fluorescent barcodes to see which drugs affected cells in different droplets [63].

## 17. Droplet Microfluidics

**Figure 10:**
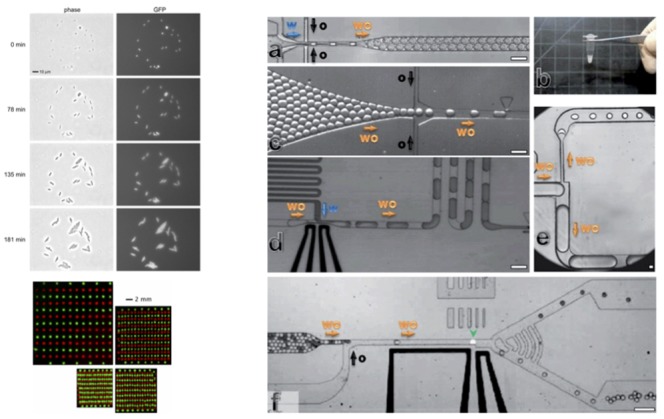
(**left**) Bacterial colony printer [54]. Cells are viable and form high-density colony arrays. (**a**) Compression of microfluidic droplets. (**b**) High throughput production of droplets. (**c**) Spacing out of droplets. (**d**) Injection into droplets. (**e**) Splitting of droplets. (**f**) High throughput fluorescence sorting of droplets [67].

The benefit of using droplets is that they act as single reaction vessels for growing cells or carrying out chemical reactions. It can be possible to enclose individual cells or molecules. Typical chemical reactions in droplets are PCR for directed evolution studies or other high throughput biological assays [64]. Typically thousands of droplets can be generated per second and sorted. Recently it has been discovered how to parallelize droplet generation on a single chip [65]. Droplets can also be merged, split, or injected using various microfluidic constructs as in Figure 10. It is possible to analyze the transcriptional profile of individual cells through a process called RNAseq using droplet microfluidics [66].

Another exciting development is the so-called millifluidic systems where one can grow cultures in larger microliter scale droplets [68]. The droplets are spaced by oil and can be addressed by a machine so one can track the growth in each droplet by moving the chain of droplets back and forth through a microscope or detector. This allows one to manage milliliters of fluid in microfluidics style experiments.

Many applications of droplet microfluidics are geared towards directed evolution. If one produces yeast cell libraries with proteins expressed on their surface, then one can test a large library of enzymes for increased functionality using droplet reaction vessels. In the most straightforward experiments of this type, one is limited by reactions that output fluorescence. One advantage of droplets is the reduction in cost and speed at which directed evolution enzymes are made [67]. For more general information about droplet microfluidics a review from David Weitz’s group is a good starting point [69].

## 18. Biotic Games

In this section, biotic games refer to games that rely upon organisms other than humans. Biotic games are mostly of interest because they get people interested in studying biology, engineering, or science. Modern society is already accustomed to games using animals through examples like polo and horse racing. Imagine classic 80s arcade games like Pacman and Pong reinvented with microfluidics. Bacteria, euglena, or paramecium act as game components [70]. Only a handful of research groups are in this field. The scientist most pushing the biotic games with microfluidics is I.H. Riedel-Kruse at Stanford. We can classify as biotic games, any type of microfluidic entertainment like micro-jukeboxes [71]. The topic of biotic games has made for some interesting conversations in the classroom, as it can lend to interesting philosophical discourse.

**Figure 11:**
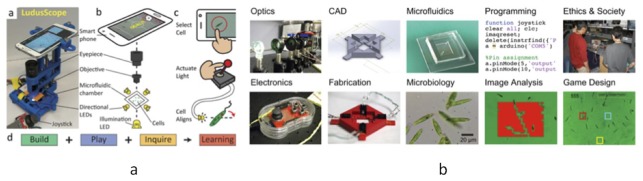
(**a**) A microfluidic game on a LudusScope [72]. (**b**) Bioengineering subject modules involving biotic games [73].

One can not predict the spinoffs of biotic games research such as the creation of new scientific instruments and technologies. Often biotic games are designed for low funded environments like museum displays as in Figure 11a, or high school classrooms as in Figure 11b. A neat example is the development of 3D printed microscope that works with standard cell phones called the LudusScope [72]. As a bioengineering course, there are a large spectrum of activities ranging from electronics, CAD, microfluidics, programming, societal ethics, optics, 3D fabrication, image analysis, and game design [73]. Interestingly, one can make topological classifications of all possible biotic games using directed graphs of three interacting objects: humans, computers, and game organisms [74].

A more scientifically relevant biotic game is the Dicty World Race [75]. The goal of this race is to understand chemotaxis and the cellular mechanisms involved. Here cells are loaded into a microfluidic maze with a chemotactic gradient and run to the exit of the labyrinth where the concentration of the gradient is highest. Anyone can compete with their choice of ameboid organisms, typically HL60 or Dictyostelium. Another exciting feature of the competition is that doping and genetic manipulations are encouraged, unlike in most other sports. The analysis of the movies makes for some fascinating insight into cell speed in gradients and choices at intersections as different strategies. Future contests may reveal even more insights into chemotaxis.

Biotic games, like any other type of research, should be evaluated ethically before their undertaking them. Many universities have strict policies about how animals can be studied or sacrificed for research. But one question remains: should there be guidelines for experimenting with non-sentient organisms like bacteria or paramecium? A recent paper considers ethical issues surrounding biotic games supporting their continuation [76]. Whether biotic games become something more popular in the lab or in science demonstrations remains to be seen.

## 19. Microfluidic Diagnostics

Microfluidics has the potential to improve global health, especially in the developing world, but there are unique constraints [77]. There may not be refrigeration for chemical reagents. Transportation may be limited, so the equipment must be lightweight. There may be a lack of electricity, clean water, and sanitation. Equipment must be able to be fine-tuned and calibrated in harsh environments. The cost of the assays must not be too expensive, perhaps in the one to five-dollar range. The devices must be easy to use because there may not be an adequate trained personnel.

The range of diseases in the developing world is much different from affluent countries. Disease range from respiratory disease, sexually transmitted diseases such as HIV, diarrhoeal diseases, malaria, measles, tuberculosis, and pertussis. There is a big leap from the laboratory proof of principle device to diagnosing people in developing countries. There is often a lack of logistics or funding to apply the technology.

The terminology point of care refers to where the doctor interacts with the patient. The microfluidic devices for point of care diagnostics may range for integrated instruments in a facility, disposable assay strips, or battery-powered hand-held devices that accept disposable cartridges. A good idea for hand-held devices is the ability to accept different cartridges for different assays. The disposable immunochromatographic test strips are currently the most successful since they are cheap and easy to use without additional equipment.

In general, point of care diagnostic instruments can be classified by which analytes they process. These usually vary from proteins, cells, nucleic acids, and metabolites. Different samples can be blood, saliva, or cell tissues. There is a recent review describing the design principles of various devices for these types of analyses [78].

## 20. Commercialization and the Future of Microfluidics

Where is microfluidics headed, and does it have a future as a disruptive technology? An example of disruptive technology is the raspberry pi computer. What you get starting at five dollars for a Pi Zero is much more powerful than the computers of 20 years ago. Scientific commercialization is now rapidly changing with the advent of competition from China. The area of 3D printing has seen a lot of competition recently. Now printers are available for several hundred dollars, whereas they cost several thousand, not a short time ago. There are now some systems to 3D print microfluidic devices, but they often lack high precision or accuracy. Technologies that have them like the Nanoscribe are costly.

Most applications of microfluidics are not disruptive enough commercially yet to drive down prices and become more widespread. For example, inventors of single-cell DNA sequencing machines or highly specialized FACS sorters may be justified in charging hundreds of thousands of dollars. Most biologists would appreciate having these capabilities, but price tags and the volume of alternative research options drives their interests and pocketbooks elsewhere. Prices only drop when the technology is disruptive enough that one absolutely must have it.

Another way to commercialize microfluidics is through a service-based approach. It is often more economical to rent a machine than to produce it yourself. One finds this trend now with DNA synthesis or next-generation sequencing. Some other proven microfluidic disruptive technologies involve blood analysis, cancer detection, or inkjet printing, to name a few.

Factors limiting microfluidic industrialization are manufacturing techniques, supply, and demand. I find the statement, “PDMS devices only cost a few dollars each” to be exaggerated. Actual costs include the barrier of entry, learning curve, photolithography, mixing equipment, plasma machines, ovens, chemical disposal, or labor required to produce the devices. A small lab might be able to process several hundred devices per week, but would be hard-pressed to implement commercial production of 10,000 or more PDMS devices in a short time scale. There may be some shortcuts like producing epoxy replicas to scale up master production and therefore multiply PDMS device construction [79].

PDMS is often appropriate for small scale basic research, but not suitable for large scale industrialization. It is often necessary to redesign chips for manufacturing as plastic cartridges or with injection molding. Manufacturing technologies may not be cost-effective or straightforward when the resolution is one micron, or the devices involve valves. Often one reads papers where microfluidics devices do ABC can potentially solve biological problems XYZ. Microfluidics literature often demonstrates that ABC works, but the followup to study XYZ never occurs in detail. There is usually no vehicle to transfer or commercialize the technology from the original lab. This void called the valley of death stymies progress in microfluidics [80].

Even if one discovered a new medicine in a lab with microfluidics, it might still take five years and a billion dollars to bring a drug to the market. Most academic labs are not a company, are not known to start up a business, or may only pursue microfluidics as a basic research technique. There have been many investigations into microfluidic diagnostic tools for low-income countries like paper-based microfluidics, but relatively few implementations in the field due to funding issues [81]. The market capitalization for microfluidics and nanotechnologies is still hundreds of billions of dollars per year and growing steadily [82]. We may now be in a transition period where microfluidics begins to have more traction as a disruptive technology.

## 21. Conclusions

This has been a wide overview of microfluidics with primarily biological applications. There are now tens of thousands of microfluidics research articles. With the references in this article, a large amount of ground can be explored in a microfluidics course even if only for a half-semester. By understanding the variety of applications of microfluidics, one can get a bigger picture of how microfluidics is influencing science. The all-encompassing lab on a chip may have not yet arrived, but there is still a lot that can be done. Microfluidics is here to stay and is at the forefront of interdisciplinary research. 

## References

[1] Folch A. (2016). Introduction to BioMEMS.

[2] Phillips R., Theriot J., Kondev J., Garcia H. (2012). Physical Biology of the Cell.

[3] Purcell E.M. (1977). Life at low Reynolds number. Am. J. Phys..

[4] Squires T.M., Quake S.R. (2005). Microfluidics: Fluid physics at the nanoliter scale. Rev. Mod. Phys..

[5] Weibel D.B., Di Luzio W.R., Whitesides G.M. (2007). Microfabrication meets microbiology. Nat. Rev. Microbiol..

[6] Xia Y., Whitesides G.M. (1998). Soft lithography. Annu. Rev. Mater. Sci..

[7] Unger M.A., Chou H.P., Thorsen T., Scherer A., Quake S.R. (2000). Monolithic microfabricated valves and pumps by multilayer soft lithography. Science.

[8] Thorsen T., Maerkl S.J., Quake S.R. (2002). Microfluidic large-scale integration. Science.

[9] Madou M.J., Microfabrication F.O. (2002). The Science of Miniaturization.

[10] Balaban N.Q., Merrin J., Chait R., Kowalik L., Leibler S. (2004). Bacterial persistence as a phenotypic switch. Science.

[11] Balagaddé F.K., You L., Hansen C.L., Arnold F.H., Quake S.R. (2005). Long-term monitoring of bacteria undergoing programmed population control in a microchemostat. Science.

[12] Wang P., Robert L., Pelletier J., Dang W.L., Taddei F., Wright A., Jun S. (2010). Robust growth of Escherichia coli. Curr. Biol..

[13] Taheri-Araghi S., Bradde S., Sauls J.T., Hill N.S., Levin P.A., Paulsson J., Vergassola M., Jun S. (2015). Cell-size control and homeostasis in bacteria. Curr. Biol..

[14] Norman T.M., Lord N.D., Paulsson J., Losick R. (2013). Memory and modularity in cell-fate decision making. Nature.

[15] Bergmiller T., Andersson A.M., Tomasek K., Balleza E., Kiviet D.J., Hauschild R., Tkacik G., Guet C.C. (2017). Biased partitioning of the multidrug efflux pump AcrAB-TolC underlies long-lived phenotypic heterogeneity. Science.

[16] Chait R., Ruess J., Bergmiller T., Tkacik G., Guet C.C. (2017). Shaping bacterial population behavior through computer interfaced control of individual cells. Nat. Commun..

[17] Moffitt J.R., Lee J.B., Cluzel P. (2012). The single-cell chemostat: An agarose-based, microfluidic device for high-throughput, single-cell studies of bacteria and bacterial communities. Lab Chip.

[18] Galajda P., Keymer J., Chaikin P., Austin R. (2007). A wall of funnels concentrates swimming bacteria. J. Bacteriol..

[19] Lambert G., Kussell E. (2014). Memory and fitness optimization of bacteria under fluctuating environments. PLoS Genet..

[20] Taniguchi Y., Choi P.J., Li G.W., Chen H., Babu M., Hearn J., Emili A., Xie X.S. (2010). Quantifying E. coli proteome and transcriptome with single-molecule sensitivity in single cells. Science.

[21] Groisman A., Lobo C., Cho H., Campbell J.K., Dufour Y.S., Stevens A.M., Levchenko A. (2005). A microfluidic chemostat for experiments with bacterial and yeast cells. Nat. Methods.

[22] Moore T.I., Chou C.S., Nie Q., Jeon N.L., Yi T.M. (2008). Robust spatial sensing of mating pheromone gradients by yeast cells. PLoS ONE.

[23] Dénervaud N., Becker J., Delgado-Gonzalo R., Damay P., Rajkumar A.S., Unser M., Shore D., Naef F., Maerkl S.J. (2013). A chemostat array enables the spatio-temporal analysis of the yeast proteome. Proc. Natl. Acad. Sci. USA.

[24] Hochstetter A., Stellamanns E., Deshpande S., Uppaluri S., Engstler M., Pfohl T. (2015). Microfluidics-based single cell analysis reveals drug-dependent motility changes in trypanosomes. Lab Chip.

[25] Stellamanns E., Uppaluri S., Hochstetter A., Heddergott N., Engstler M., Pfohl T. (2014). Optical trapping reveals propulsion forces, power generation and motility efficiency of the unicellular parasites Trypanosoma brucei brucei. Sci. Rep..

[26] Holm S.H., Beech J.P., Barrett M.P., Tegenfeldt J.O. (2016). Simplifying microfluidic separation devices towards field-detection of blood parasites. Anal. Methods.

[27] McGrath J., Jimenez M., Bridle H. (2014). Deterministic lateral displacement for particle separation: A review. Lab Chip.

[28] Salafi T., Zhang Y., Zhang Y. (2019). A Review on Deterministic Lateral Displacement for Particle Separation and Detection. Nano Micro Lett..

[29] Nakagaki T., Yamada H., Tóth Á. (2000). Intelligence: Maze-solving by an amoeboid organism. Nature.

[30] Tero A., Takagi S., Saigusa T., Ito K., Bebber D.P., Fricker M.D., Yumiki K., Kobayashi R., Nakagaki T. (2010). Rules for biologically inspired adaptive network design. Science.

[31] Gray J.M., Karow D.S., Lu H., Chang A.J., Chang J.S., Ellis R.E., Marletta M.A., Bargmann C.I. (2004). Oxygen sensation and social feeding mediated by a C. elegans guanylate cyclase homologue. Nature.

[32] Chronis N., Zimmer M., Bargmann C.I. (2007). Microfluidics for in vivo imaging of neuronal and behavioral activity in Caenorhabditis elegans. Nat. Methods.

[33] Rohde C.B., Zeng F., Gonzalez-Rubio R., Angel M., Yanik M.F. (2007). Microfluidic system for on-chip high-throughput whole-animal sorting and screening at subcellular resolution. Proc. Natl. Acad. Sci. USA.

[34] Hulme S.E., Shevkoplyas S.S., McGuigan A.P., Apfeld J., Fontana W., Whitesides G.M. (2010). Lifespan-on-a-chip: Microfluidic chambers for performing lifelong observation of C. elegans. Lab Chip.

[35] Kamili F., Lu H. (2018). Recent advances and trends in microfluidic platforms for C. elegans biological assays. Annu. Rev. Anal. Chem..

[36] Lucchetta E.M., Lee J.H., Fu L.A., Patel N.H., Ismagilov R.F. (2005). Dynamics of Drosophila embryonic patterning network perturbed in space and time using microfluidics. Nature.

[37] Ghannad-Rezaie M., Wang X., Mishra B., Collins C., Chronis N. (2012). Microfluidic chips for in vivo imaging of cellular responses to neural injury in Drosophila larvae. PLoS ONE.

[38] Chen C.C., Zappe S., Sahin O., Zhang X.J., Fish M., Scott M., Solgaard O. (2004). Design and operation of a microfluidic sorter for Drosophila embryos. Sens. Actuators Chem..

[39] Grossmann G., Guo W.J., Ehrhardt D.W., Frommer W.B., Sit R.V., Quake S.R., Meier M. (2011). The RootChip: An integrated microfluidic chip for plant science. Plant Cell.

[40] Fendrych M., Akhmanova M., Merrin J., Glanc M., Hagihara S., Takahashi K., Uchida N., Torii K.U., Friml J. (2018). Rapid and reversible root growth inhibition by TIR1 auxin signalling. Nat. Plants.

[41] Nezhad A.S. (2014). Microfluidic platforms for plant cells studies. Lab Chip.

[42] Stanley C.E., Shrivastava J., Brugman R., Heinzelmann E., van Swaay D., Grossmann G. (2018). Dual-flow-RootChip reveals local adaptations of roots towards environmental asymmetry at the physiological and genetic levels. New Phytol..

[43] Haessler U., Kalinin Y., Swartz M.A., Wu M. (2009). An agarose-based microfluidic platform with a gradient buffer for 3D chemotaxis studies. Biomed. Microdevices.

[44] Schwarz J., Bierbaum V., Merrin J., Frank T., Hauschild R., Bollenbach T., Tay S., Sixt M., Mehling M. (2016). A microfluidic device for measuring cell migration towards substrate-bound and soluble chemokine gradients. Sci. Rep..

[45] Love J.C., Ronan J.L., Grotenbreg G.M., van der Veen A.G., Ploegh H.L. (2006). A microengraving method for rapid selection of single cells producing antigen-specific antibodies. Nat. Biotechnol..

[46] Wang T., Zhang M., Dreher D.D., Zeng Y. (2013). Ultrasensitive microfluidic solid-phase ELISA using an actuatable microwell-patterned PDMS chip. Lab Chip.

[47] Renkawitz J., Kopf A., Stopp J., de Vries I., Driscoll M.K., Merrin J., Hauschild R., Welf E.S., Danuser G., Fiolka R. (2019). Nuclear positioning facilitates amoeboid migration along the path of least resistance. Nature.

[48] Lee H., Lee B.P., Messersmith P.B. (2007). A reversible wet/dry adhesive inspired by mussels and geckos. Nature.

[49] Baik S., Park Y., Lee T.J., Bhang S.H., Pang C. (2017). A wet-tolerant adhesive patch inspired by protuberances in suction cups of octopi. Nature.

[50] Skardal A., Shupe T., Atala A. (2016). Organoid-on-a-chip and body-on-a-chip systems for drug screening and disease modeling. Drug Discov. Today.

[51] Shuler M.L. (2017). Organ-, body- and disease-on-a-chip systems. Lab Chip.

[52] Ramadan Q., Alberti M., Dufva M., Tung Y.C. (2019). Medical and Industrial Applications of Microfluidic-based Cell/Tissue Culture and Organs-on-a-Chip. Front. Bioeng. Biotechnol..

[53] Huh D., Matthews B.D., Mammoto A., Montoya-Zavala M., Hsin H.Y., Ingber D.E. (2010). Reconstituting organ-level lung functions on a chip. Science.

[54] Merrin J., Leibler S., Chuang J.S. (2007). Printing multistrain bacterial patterns with a piezoelectric inkjet printer. PLoS ONE.

[55] Lausted C., Dahl T., Warren C., King K., Smith K., Johnson M., Saleem R., Aitchison J., Hood L., Lasky S.R. (2004). POSaM: A fast, flexible, open-source, inkjet oligonucleotide synthesizer and microarrayer. Genome Biol..

[56] Tian J., Ma K., Saaem I. (2009). Advancing high-throughput gene synthesis technology. Mol. Biosyst..

[57] Quan J., Saaem I., Tang N., Ma S., Negre N., Gong H., White K.P., Tian J. (2011). Parallel on-chip gene synthesis and application to optimization of protein expression. Nat. Biotechnol..

[58] Karzbrun E., Tayar A.M., Noireaux V., Bar-Ziv R.H. (2014). Programmable on-chip DNA compartments as artificial cells. Science.

[59] Heymann M., Opthalage A., Wierman J.L., Akella S., Szebenyi D.M., Gruner S.M., Fraden S. (2014). Room-temperature serial crystallography using a kinetically optimized microfluidic device for protein crystallization and on-chip X-ray diffraction. IUCrJ.

[60] Heyries K.A., Tropini C., VanInsberghe M., Doolin C., Petriv I., Singhal A., Leung K., Hughesman C.B., Hansen C.L. (2011). Megapixel digital PCR. Nat. Methods.

[61] Huang C.J., Lin H.I., Shiesh S.C., Lee G.B. (2012). An integrated microfluidic system for rapid screening of alpha-fetoprotein-specific aptamers. Biosens. Bioelectron..

[62] Feng X., Liu B.F., Li J., Liu X. (2015). Advances in coupling microfluidic chips to mass spectrometry. Mass Spectrom. Rev..

[63] Brouzes E., Medkova M., Savenelli N., Marran D., Twardowski M., Hutchison J.B., Rothberg J.M., Link D.R., Perrimon N., Samuels M.L. (2009). Droplet microfluidic technology for single-cell high-throughput screening. Proc. Natl. Acad. Sci. USA.

[64] Guo M.T., Rotem A., Heyman J.A., Weitz D.A. (2012). Droplet microfluidics for high-throughput biological assays. Lab Chip.

[65] Amstad E., Chemama M., Eggersdorfer M., Arriaga L.R., Brenner M.P., Weitz D.A. (2016). Robust scalable high throughput production of monodisperse drops. Lab Chip.

[66] Macosko E.Z., Basu A., Satija R., Nemesh J., Shekhar K., Goldman M., Tirosh I., Bialas A.R., Kamitaki N., Martersteck E.M. (2015). Highly parallel genome-wide expression profiling of individual cells using nanoliter droplets. Cell.

[67] Agresti J.J., Antipov E., Abate A.R., Ahn K., Rowat A.C., Baret J.C., Marquez M., Klibanov A.M., Griffiths A.D., Weitz D.A. (2010). Ultrahigh-throughput screening in drop-based microfluidics for directed evolution. Proc. Natl. Acad. Sci. USA.

[68] Baraban L., Bertholle F., Salverda M.L., Bremond N., Panizza P., Baudry J., de Visser J.A.G., Bibette J. (2011). Millifluidic droplet analyser for microbiology. Lab Chip.

[69] Mashaghi S., Abbaspourrad A., Weitz D.A., van Oijen A.M. (2016). Droplet microfluidics: A tool for biology, chemistry and nanotechnology. TrAC Trends Anal. Chem..

[70] Riedel-Kruse I.H., Chung A.M., Dura B., Hamilton A.L., Lee B.C. (2011). Design, engineering and utility of biotic games. Lab Chip.

[71] Tan S.H., Maes F., Semin B., Vrignon J., Baret J.C. (2014). The microfluidic jukebox. Sci. Rep..

[72] Kim H., Gerber L.C., Chiu D., Lee S.A., Cira N.J., Xia S.Y., Riedel-Kruse I.H. (2016). LudusScope: Accessible interactive smartphone microscopy for life-science education. PLoS ONE.

[73] Cira N.J., Chung A.M., Denisin A.K., Rensi S., Sanchez G.N., Quake S.R., Riedel-Kruse I.H. (2015). A biotic game design project for integrated life science and engineering education. PLoS Biol..

[74] Gerber L.C., Kim H., Riedel-Kruse I.H. (2016). Interactive Biotechnology: Design Rules for Integrating Biological Matter into Digital Games. DiGRA/FDG.

[75] Skoge M., Wong E., Hamza B., Bae A., Martel J., Kataria R., Keizer-Gunnink I., Kortholt A., Van Haastert P.J., Charras G. (2016). A worldwide competition to compare the speed and chemotactic accuracy of neutrophil-like cells. PLoS ONE.

[76] Harvey H., Havard M., Magnus D., Cho M.K., Riedel-Kruse I.H. (2014). Innocent Fun or “Microslavery”? AN ETHICAL ANALYSIS OF BIOTIC GAMES. Hastings Cent. Rep..

[77] Yager P., Edwards T., Fu E., Helton K., Nelson K., Tam M.R., Weigl B.H. (2006). Microfluidic diagnostic technologies for global public health. Nature.

[78] Jung W., Han J., Choi J.W., Ahn C.H. (2015). Point-of-care testing (POCT) diagnostic systems using microfluidic lab-on-a-chip technologies. Microelectron. Eng..

[79] Kamande J.W., Wang Y., Taylor A.M. (2015). Cloning SU8 silicon masters using epoxy resins to increase feature replicability and production for cell culture devices. Biomicrofluidics.

[80] Islam N. (2017). Crossing the Valley of Death-an Integrated Framework and a Value Chain for Emerging Technologies. IEEE Trans. Eng. Manag..

[81] Martinez A.W., Phillips S.T., Whitesides G.M., Carrilho E. (2010). Diagnostics for the developing world: Microfluidic paper-based analytical devices. Anal. Chem..

[82] Alvarez M.M., Aizenberg J., Analoui M., Andrews A.M., Bisker G., Boyden E.S., Kamm R.D., Karp J.M., Mooney D.J., Oklu R. (2017). Emerging trends in micro-and nanoscale technologies in medicine: From basic discoveries to translation. ACS Nano.

